# Blood Drain: Soil-Transmitted Helminths and Anemia in Pregnant Women

**DOI:** 10.1371/journal.pntd.0002912

**Published:** 2014-07-10

**Authors:** Theresa W. Gyorkos, Nicolas L. Gilbert

**Affiliations:** 1 Parasite Epidemiology Research Group, Division of Clinical Epidemiology, Research Institute of the McGill University Health Centre, Montreal, Canada; 2 Department of Epidemiology, Biostatistics and Occupational Health, McGill University, Montreal, Canada; Ministère de la Santé Publique et de la Lutte contre les Endémies, Niger

## Anemia and Maternal Health

The main cause of anemia is iron deficiency, which, in turn, can result from a wide range of factors. Iron intake may be insufficient because of a low dietary iron content, less bioavailable (i.e., non-heme) iron, or because of poor absorption of iron due to concurrent ingestion of inhibitors such as cereals and grain [1]. In addition, iron may be lost because of infection by parasites that destroy red blood cells, such as malaria-causing *Plasmodium* species, or by blood-feeding parasites, such as the soil-transmitted helminths (STHs) [1].

Normal hemoglobin distributions vary by age and sex and so do anemia thresholds (i.e., hemoglobin (Hb) concentrations below which individuals are considered anemic). Among women of reproductive age, this threshold is 11 g/dL for pregnant women and 12 g/dL for nonpregnant women [2]. Anemia in pregnant women is of particular concern because of its association with maternal mortality. This association has been known for years [3], [4] and has been confirmed by recent evidence from cohort studies [5], [6]. The World Health Organization estimates that the worldwide prevalence of anemia is 30% in pregnant women and 20% in nonpregnant women, with higher levels in women living in developing countries [7]. The estimated prevalence of anemia in pregnant women is highest in Africa (55.8%) and Asia (41.6%) and lowest in North America (6.1%) and Europe (18.7%) [7].

## The Evidence Linking Soil-Transmitted Helminths and Anemia

The association between hookworm infection and anemia is well known. Hookworm infection is recognized as a risk factor for anemia, both in pregnant women [8] and in nonpregnant women [9]. In pregnant women, a meta-analysis published in 2008 showed that even light intensity (1–1,999 eggs per gram [epg]) hookworm infection is associated with a significant decrease in blood Hb, and that the magnitude of the Hb decrement increases with infection intensity [8]. Since then, new studies have provided further evidence documenting the detrimental effect of hookworm infection on Hb levels [10], [11].

The evidence linking the whipworm, *Trichuris trichiura*, with anemia is less clear. Most studies of Hb/anemia in pregnant women compared *Trichuris*-infected and non-*Trichuris*-infected women, and found no statistically significant associations [11]–[15]. However, none of these studies had differentiated between *Trichuris* infection intensity categories. Of the single study which had examined *Trichuris* infection intensity categories, results showed that pregnant women with moderate or heavy *Trichuris* infection in their second trimester of pregnancy had statistically significantly lower Hb levels compared to those with no or light *Trichuris* infection, despite all of the women receiving iron supplementation [16]. It, therefore, showed an association between both *Trichuris* infection and increasing *Trichuris* intensity, as well as an increased risk of anemia—and this despite iron supplementation.

Moreover, there is limited evidence demonstrating that, in cases where there is co-infection of *Trichuris* with hookworm, the risk of anemia increases with increasing intensity of either *Trichuris* infection or hookworm infection [16]. However, more research is needed on this topic.

Similar patterns have been observed in children. Some studies have shown an association between *Trichuris* infection and lower Hb levels, or anemia [17]–[19], while others have not [20]–[22]. However, there are major differences between these studies with respect to endemicity levels and analytical approach. For instance, most studies that showed no association were conducted in populations where the low prevalence or the intensity distribution of *Trichuris* infection precluded analyses by infection intensity categories. In contrast, studies that showed an association between *Trichuris* and lower Hb levels included sufficient numbers of infected subjects to divide them into intensity categories, and found associations between higher infection intensity categories and lower Hb levels [17], [18].

## STH Prevention and Control Strategies

The effectiveness of anthelminthic treatment to reduce anemia during pregnancy has been examined in three randomized controlled trials (RCTs) to date. These were, in turn, examined in two systematic reviews and meta-analyses, concluding that anthelminthics do not significantly increase Hb levels or decrease the prevalence of anemia during pregnancy [23], [24].

The apparent lack of deworming effectiveness can be explained, in part, by the low prevalence of STH infection in two of the three study populations. Consequently, these studies likely had insufficient power to detect a statistically significant effect, if one existed. Another partial explanation may be the limited effectiveness of anthelminthics against *T. trichiura*. Indeed, a recent meta-analysis of RCTs showed that commonly used single-dose treatments with albendazole (400 mg) or mebendazole (500 mg) are of limited efficacy against *Trichuris* infection, while albendazole is more effective than mebendazole against hookworms [25].

Anthelminthic agents, while essential in endemic areas, may not be sufficient, alone, to achieve sustained elimination of STHs and, consequently, of related STH-attributable anemia. This suggests a need for a shift “from morbidity control to transmission control” [26]. For example, the role of health education, integrated into an overall intervention strategy, and targeted to specific healthcare personnel and population subgroups, has been shown to be effective in reducing both the prevalence of hookworm infection and anemia [27]. Similarly, a recent systematic review has shown that adequate waste management and sanitation (defined as access and use of facilities for the safe disposal of human urine and feces) is associated with reduced transmission of *Ascaris lumbricoides*, *T. trichiura*, and hookworms [28].

It is clear that a sustainable reduction in STH-attributable maternal anemia would benefit from both short-term and long-term prevention and control strategies. Efforts to address the Millennium Development Goals (MDGs), in particular MDG 5 (improve maternal health) and MDG 7 (e.g., Target 7C: halve, by 2015, the proportion of people without sustainable access to safe drinking water and basic sanitation), would contribute to achieving such a reduction. With global attention increasingly focused on alleviating the inequities resulting from Neglected Tropical Diseases, now is the time to ensure an evidence-informed, comprehensive, and integrated strategy to both deworm infected individuals and prevent infections by deploying sanitation and hygiene interventions.
